# Donkey-derived anti-CDV IgG, as a passive immunotherapy agent, can effectively increase survival rates of the experimental CDV-infected dogs

**DOI:** 10.1186/s12917-021-02982-y

**Published:** 2021-08-06

**Authors:** Jianlou Zhang, Dan Cui, Yuzhu Zuo, Zhiqiang Zheng, Fengyang Wu, Wenyan Li, Yonghong Zhang, Shanshan Huo, Nan Li, Lanhui Li, Yueqiang Guan, Fei Zhong

**Affiliations:** 1 School of Veterinary Medicine, Hebei Veterinary Biotechnology Innovation Center, Hebei Agricultural University, 289 Lingyusi Streat, Baoding, 071001 Hebei China; 2 School of Animal Science and Technology, Hebei Agricultural University, 289 Lingyusi Streat, Baoding, 071001 Hebei China; 3 School of Basic Medicine, Hebei University, 342 Yuhua East Road, Baoding, 071002 Hebei China; 4 School of Life Science, Hebei University, 180 Wusi East Road, Baoding, 071002 Hebei China

**Keywords:** Canine distemper virus, Passive immunotherapy, Heterologous antibody, Donkey, Therapeutic effects

## Abstract

**Background:**

Humoral immunity plays an important role in the prevention of canine distemper. Anti-CD virus (CDV) antibody has strong antiviral activity and is widely used in the treatment of CD. However, with the increase of CD cases, the availability of therapeutic CD antibody fell short of the clinical needs.

**Results:**

The high-titer antiserum with the high-titer neutralizing activity against CDV was obtained from the donkeys (Dezhou Donkey) immunized with the inactivated CDV vaccine. The donkey anti-CDV IgG was purified from the donkey serum, which was identified to significantly inhibit the CDV replication in the cultured Vero cells and effectively reduce the clinical symptoms and increase the survival rates (75%) of CDV-infected dogs (Shih-tzu Dog), similar to that treated with the dog-derived anti-CDV IgG. These results indicate that donkey-derived IgG is a potential substitute for dog-derived IgG to treat the CD in clinic.

**Conclusions:**

Administration of donkey-derived anti-CDV IgG can ameliorate clinical symptoms and inhibit virus replication, thereby increasing the survival of CDV-infected dogs. This study opens up a new source of therapeutic antibody for CD treatment.

## Background

Canine distemper (CD) is an acute, highly contagious disease caused by canine distemper virus (CDV) in canines, clinically characterized by increased biphasic body temperature, subsequent bronchitis, catarrhal pneumonia, severe gastroenteritis and neurological symptoms. Eventually the infected dogs often die because of central nervous system damage [1–3]. CD has high morbidity and mortality rates, the mortality rate is over 90% in infected puppies aged from 2 to 5 months [4, 5]. CDV has a wide range of hosts, infecting not only dogs of all ages but also ferrets, raccoons and giant animals of many species, such as giant pandas, tigers, lions, pandas, lynx and bears [6, 7]. The epidemic of CD often results in huge economic loss to the dog industry, economic animal breeding industry and wildlife protection, as well as a certain mental pressure on pet owners [8, 9]. Therefore, effective prevention and treatment of CD is urgent and of vital importance.

Prevention of CD is known primarily through inoculating vaccination against CDV [10]. However, due to the lack of highly effective vaccine and the interference of maternal antibody, the immunized puppies are not 100% protected from CDV infection. Therefore, CD has become a common infectious disease in pet clinic [11]. Due to its excellent therapeutic effect, the most effective anti-CDV drug remains to be CDV antiserum (or antibody) that conveys satisfactory prevention and treatment of CD [12]. Currently, the antiserum or antibody used to prevent/treat CD was mainly derived from dogs (homologous animals) [13, 14]. However, due to the shortage of dogs and the vast majority of dogs dispersed in the dog owners, the antiserum and antibody produced by the limited dog farms could not meet the clinical needs. Additionally, the cost of producing dog antibody is high, thereby negatively impacting the clinical application of anti-CDV antibodies [15]. It is therefore necessary to develop antiserum or antibody from the heterologous animals, especially from larger heterologous animals to solve the problem of shortage of CDV antibody for common pet animals (such as dogs, cats) or rare wild animals (tigers, leopards, monkeys, etc.) in zoos.

The larger animals used to prepare heterologous antibodies are usually equine animals, especially horses [16–18]. Horses have a long history as heterologous animals being used to prepare antibodies for the prevention and treatment of other animal diseases [19]. For instance, horse-derived immunoglobulin F (ab´)2 were used to neutralize a large panel of highly pathogenic avian influenza A viruses (H5N1) [20, 21], to treat Middle East respiratory syndrome coronavirus infection in a mouse models [22, 23], to treat experimental Ebola virus infection [24, 25] and to treat severe respiratory syndrome-associated coronavirus infection [26–28]. Donkeys belong to equine animals, which are relatively resistant to developing diseases, such as quine infectious anemia and other infectious diseases [29]. Moreover, relative to other animals, donkeys tend to mount stronger antigen-specific immune responses, making them the most common equine animals in which biological reagents are produced [30]. Although donkeys are prevalent in China, especially in the rural areas of Northern China, very few therapeutic antibodies were prepared from the donkeys. This may be related to the fact that the use of horse-derived antibodies for therapeutic purposes has a long history, and their preparation methods are relatively mature [31]. To explore alternative sources of therapeutic antibodies that can mitigate the shortage of anti-CDV antibody, in this study we used donkeys to prepare therapeutic anti-CDV antibody, and analyze its therapeutic effects and safety. This study provides a new source of antibody for CD therapy.

## Results

### Donkeys have a strong humoral response to inactivated CDV vaccine

**Fig. 1:**
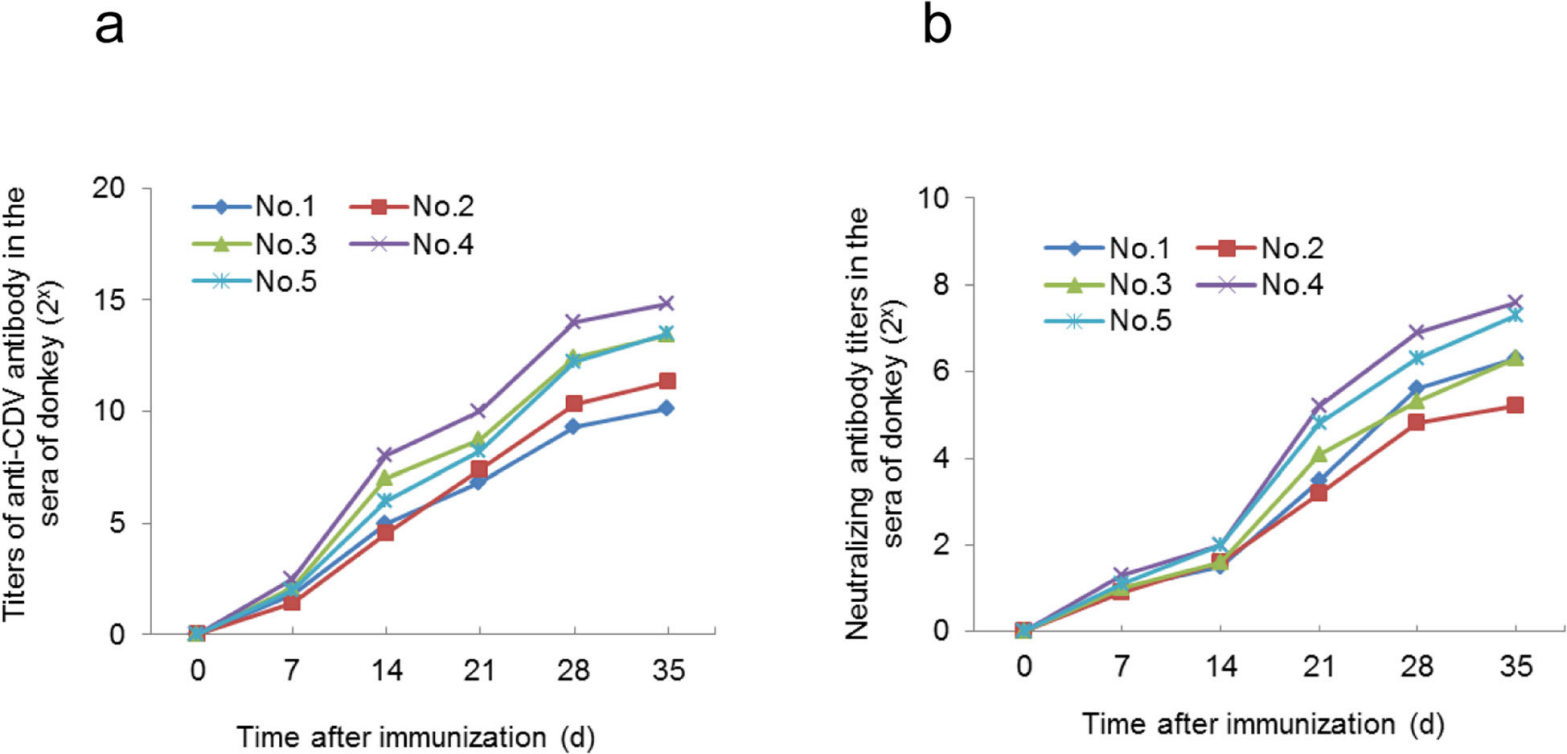
Titers of antibody and neutralizing antibody against CDV in the sera of donkeys immunized with inactivated CDV vaccine at the different time after immunization. (a) Titers of antibody measured with ELISA. (b) Titers of neutralizing antibody measured with micro neutralization test

To quantify the humoral immune response against CDV, donkeys were inoculated with inactivated CDV vaccine and the titers of CDV-specific antibodies in the serum of donkeys were measured by enzyme-linked immunosorbent assay (ELISA) at different times after immunization (Fig. 1a). We found that CDV-specific antibodies emerges at 7 d after immunization in all donkey sera. However, the antibody titer was relatively low, about 2^2^ ~ 2^3^. From the 7th to the 28th day after immunization, the titer of antibody increased sharply within the duration of immunization, from average titer of 2^5^ at 7 d to 2^12^ at 28 d. After 28 d of immunization, the titer of antibody increased slowly, and the titer of antibody was about 2^13^ at 35 d. It also can be seen from Fig. 1a that the humoral immune responses of donkeys to CDV inactivated vaccine vary significantly in different individuals. In general, our results indicate that donkey has a rapid and strong humoral response to inactivated CDV vaccine, and the high-titer antibody against CDV can be achieved in immunized donkeys 1 month after immunization.

In order to determine the neutralizing activity of CDV-specific antibody, the titer of neutralizing antibody in sera of the immunized donkeys was detected by micro-neutralization test at different time points after immunization (Fig. 1b). We found that the neutralizing antibody titers tended to be consistent with the total antibody titer during the immunization periods. The neutralizing antibody titer began to rise at 7 d after immunization, which rose sharply at 14 d and reached 2^6^–2^8^ at 35 d after immunization (Fig. 1b).

### Anti-CDV IgG prepared from donkey sera has the potent antiviral activity against CDV in vitro

**Fig. 2:**
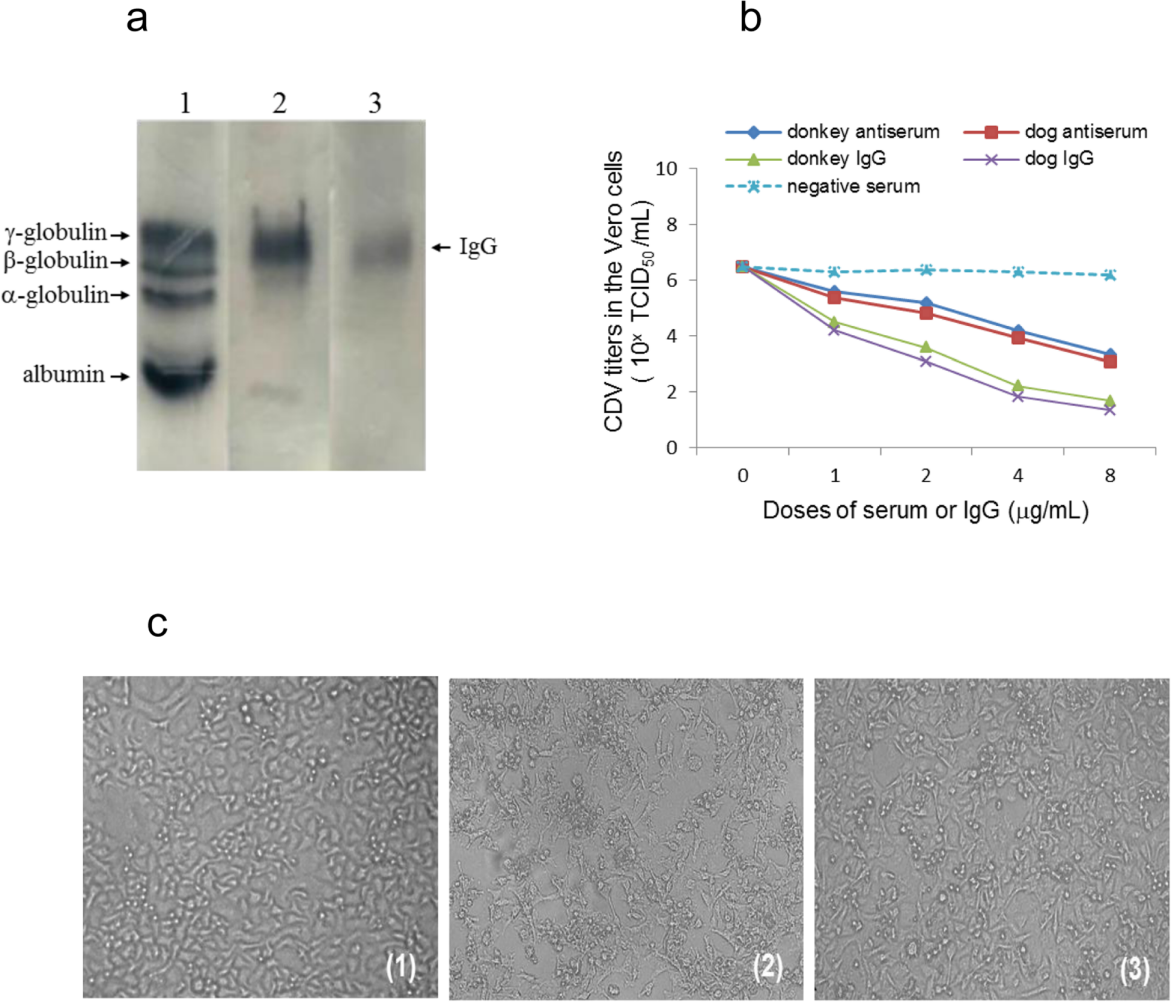
Purification of donkey IgG and its anti-CDV activity in cultured Vero cells. (a) Cellulose acetate film electrophoresis for donkey serum and purified IgG. 1 ~ 3, serum, crude IgG by salting out, and purified IgG with ion-exchange chromatography. (b) Comparison of anti-CDV activity of the serum and IgG derived from donkeys and dogs at the different doses. (c) Morphology of Vero cells under different treatments for 72 h. (1) un-treated cells as a control; (2) CDV-infected cells; (3) IgG-pretreated CDV-infected cells

The main hurdle of passive immunotherapy using heterologous antiserum is a phenomenon called immune rejection, which not only reduces the therapeutic effect, but also produces toxic and side effects [32]. To limit immune rejection in dogs against donkey sera, the anti-CDV IgG should be isolated and purified from donkey serum. We therefore isolated and purified IgG from donkey serum by salting out and ion exchange chromatography, and obtained IgG with high purity of ~ 98% (Fig. 2a).

The above experiment has confirmed that the neutralizing antibody titer of donkey serum is 2^6^, indicating that the donkey antiserum possesses high anti-CDV activity. To further confirm the anti-CDV activity of the IgG purified from donkey serum in vitro, and compare its anti-CDV activity with dog IgG, Vero cells were used to analyze the anti-CDV activity. Our results showed that both antisera and purified IgG could reduce CDV titers in a dose-dependent manner (Fig. 2b). The antiviral activity of donkey anti-serum and IgG was similar to that of dog anti-serum and IgG, indicating that there was no significant difference in antiviral activity between donkey and dog antiserum and IgG. By comparing the antiviral ability between donkey anti-serum and donkey IgG, it was confirmed that the donkey IgG showed the higher antiviral ability than donkey antiserum with similar doses (Fig. 2b).

In order to further confirm the anti-CDV activity of donkey IgG in vitro, we analyzed the pathological changes of Vero cells after CDV infection and the inhibitory effect of donkey IgG on CDV-induced lesions (Fig. 2c). Our results suggest that serious pathological changes appeared in Vero cells at 72 h after CDV infection (Fig. 2c2), while the CDV-induced lesions could be significantly alleviated in the presence of donkey IgG (Fig. 2c3), further supporting that donkey IgG has obvious anti-CDV effect in vitro.

### Donkey IgG increases the survival rates of CDV-infected dogs

**Fig. 3:**
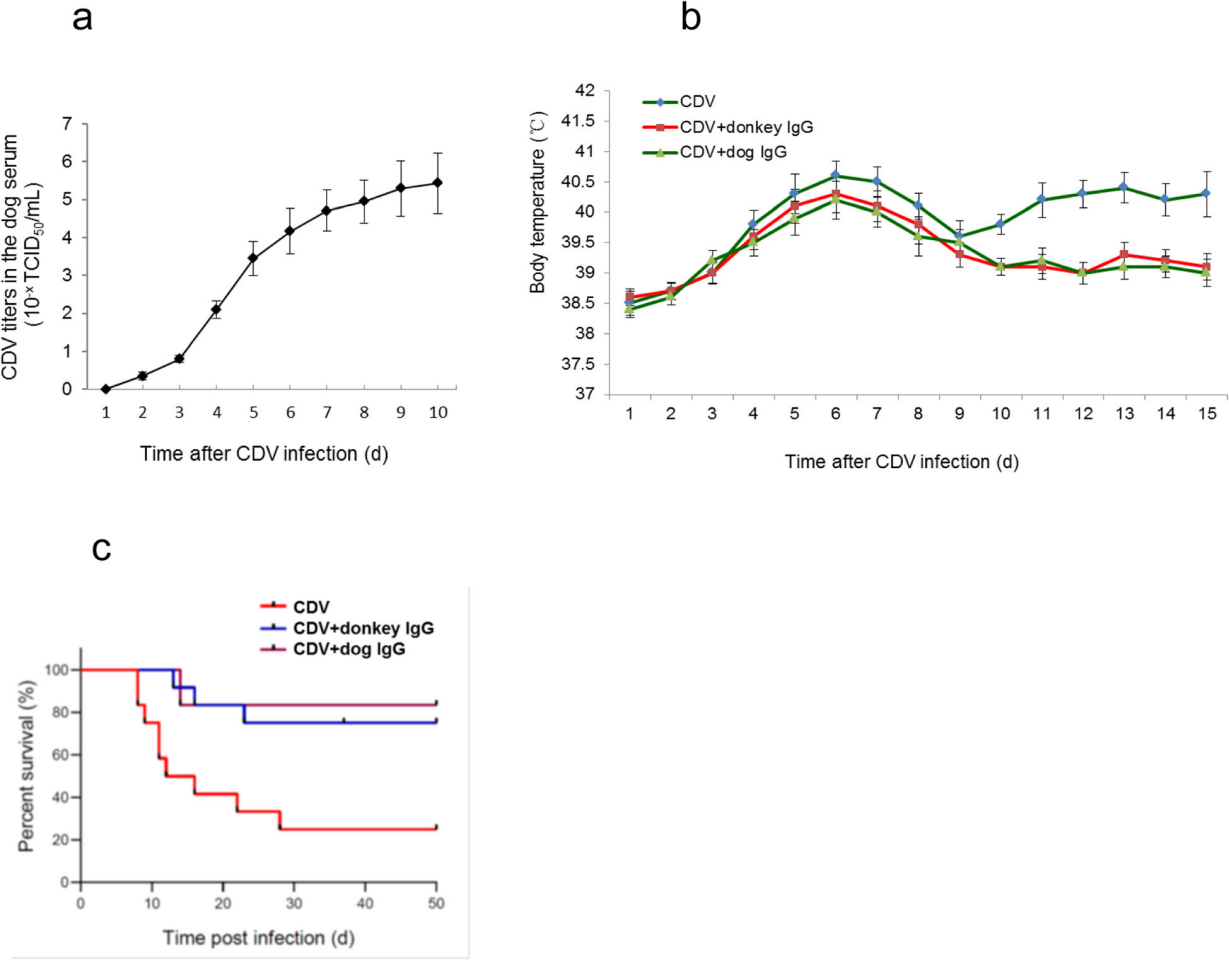
Therapeutic effects of donkey-derived IgG on CDV-infected dogs. (a) CDV titers in the serum of dogs after CDV infection at the different time. (b) Effects of donkey-derived IgG on the body temperature of CDV-infected dogs. (c) Effects of donkey-derived IgG on the survival rates of CDV-infected dogs

To analyze the therapeutic effect of donkey IgG on the experimental CD, the CDV titer in the serum of CDV-infected dogs was first measured to understand the dynamics of the virus in the blood so as to determine the timing of treatment. The results showed that CDV could be detected 3–4 days after infection, and increased rapidly 5 days after infection, and the rate of virus titer increase significantly decreased at 7–8 d after infection, but the virus titer remained at a high level (Fig. 3a).

Based on the dynamic change of CDV titers in the blood of CDV-infected dog, three groups (CDV, CDV + Donkey IgG and CDV + dog IgG) of dogs (12 × 3 = 36) were infected with CDV (3 ml of 10^6^ TCID_50_). The CDV + Donkey IgG and CDV + dog IgG groups of dogs were treated with donkey IgG and dog IgG, respectively, 4 d after infection. In contrast, the CDV group was treated with negative donkey control IgG. The body temperature was measured, and the clinical symptoms were observed and the survival rates of the dogs were recorded during the experimental period.

We found that without IgG treatment, the dog body temperature in the CDV group began to rise 2–3 days after CDV infection, and reached more than 40 °C after 5–6 days of infection, and then dropped close to normal temperature (39.5 °C) 9 days post infection (Fig. 3b). Compared with the CDV group, the body temperature of the dogs in the IgG treated groups, either donkey- or dog-derived IgG were significantly different. At 2–3 days after infection, the body temperature of the dogs in the IgG treated groups were similar to that in the CDV group, and the temperature change observed in the IgG treated groups were similar to that in the CDV group, but lower than that in the CDV group at 5–9 days after infection. After 10 days of infection, the body temperature of the infected dogs in the CDV group rose again and lasted until the death of the animals. However, the body temperature of the dogs in the IgG treated groups decreased gradually and were close to the normal temperature (Fig. 3b), indicating that IgG treatments could inhibit the increase of body temperature caused by CDV infection. The results also showed that there was no significant difference of the body temperature between donkey IgG- and dog IgG-treated dogs (Fig. 3b).

Table 1 shows the severity of the clinical symptoms of the three groups of dogs after CDV infection and IgG treatment. It can be seen that the main clinical symptoms, such as loss of appetite, dropsy, runny nose and cough, were extremely serious in the CDV group for 5–6 days, while the above symptoms in the two IgG-treated groups were milder than those in the CDV group. After 11–15 days post CDV infection, the above symptoms in the CDV group were further aggravated, accompanied by severe diarrhea, dehydration and neurological symptoms. However, in the IgG-treated groups, these symptoms were significantly relieved or even disappeared after 11–15 days of infection, without diarrhea, dehydration and neurological complications. Meanwhile, there is no significant differences between donkey- and dog-derived IgGs in regards to relieving the above mentioned symptoms. These results together indicate that donkey-derived IgG has similar capacity as dog-derived IgG in terms of alleviating the clinical manifestations of CDV infected dogs.

**Table 1:** Effects of donkey-derived IgG on clinical symptoms of the CDV-infected dogs at the different time

Symptoms	Time after CDV infection (d)														
	CDV					CDV + Donkey IgG					CDV + Dog IgG				
	2–3	5–6	8–9	11–12	14–15	2–3	5–6	8–9	11–12	14–15	2–3	5–6	8–9	11–12	14–15
Loss of appetite	**+**	**+++**	**++**	**+++**	**+++**	**+**	**++**	**++**	**++**	**+**	**+**	**++**	**++**	**+**	**+**
Gum in the eyes	**–**	**+++**	**++**	**+++**	**+++**	**–**	**++**	**+**	**+**	**–**	**–**	**+**	**+**	**+**	**–**
Running at the nose	**–**	**+++**	**++**	**+++**	**+++**	**–**	**++**	**+**	**+**	**–**	**–**	**++**	**+**	**+**	**–**
Cough	**–**	**++**	**+**	**+++**	**+++**	**–**	**+**	**+**	**+**	**–**	**–**	**+**	**+**	**–**	**–**
Diarrhea/dehydration	**–**	**++**	**+**	**+++**	**+++**	**–**	**+**	**+**	**–**	**–**	**–**	**–**	**+**	**–**	**–**
Neurologic symptom	**–**	**–**	**–**	**++**	**+++**	**–**	**–**	**–**	**–**	**–**	**–**	**–**	**–**	**–**	**–**

+, ++ and +++ respectively represent the severity of symptoms: mild, moderate and severe

Survival rate of CDV-infected dogs can directly reflect the therapeutic effect of donkey IgG. Without administration with the IgG, the survival rate of CDV-infected dogs was 25% (3/12), which is significantly lower than that of the IgG-treated dogs (*P* < 0.01) (Fig. 3c). However, there was no significant difference (*P* > 0.05) in the survival rate of the dogs between CDV + donkey IgG group (75%, 9/12) and CDV + dog IgG group (83%, 10/12) (Fig. 3c). These results suggest that the donkey IgG possesses potent anti-CDV activity in vivo, thereby confirming that donkey-derived anti-CDV IgG can enhance survival of CDV-infected dogs. Last but not least, it should be noted that the therapeutic effect of donkey IgG on CDV-infected dogs is similar to that of dog IgG (Fig. 3c), suggesting that donkey IgG is a good alternative of dog IgG in clinical application.

### Donkey IgG potently inhibits CDV replication in CDV-infected dogs

**Fig. 4:**
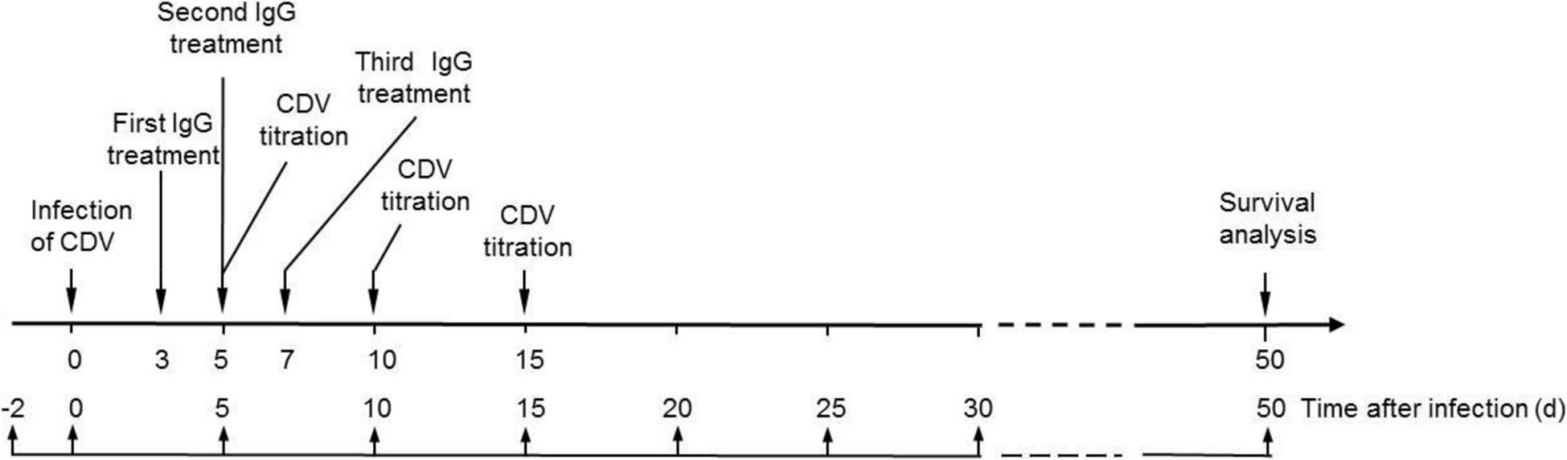
Timeline of CDV infection and IgG treatment

**Fig. 5:**
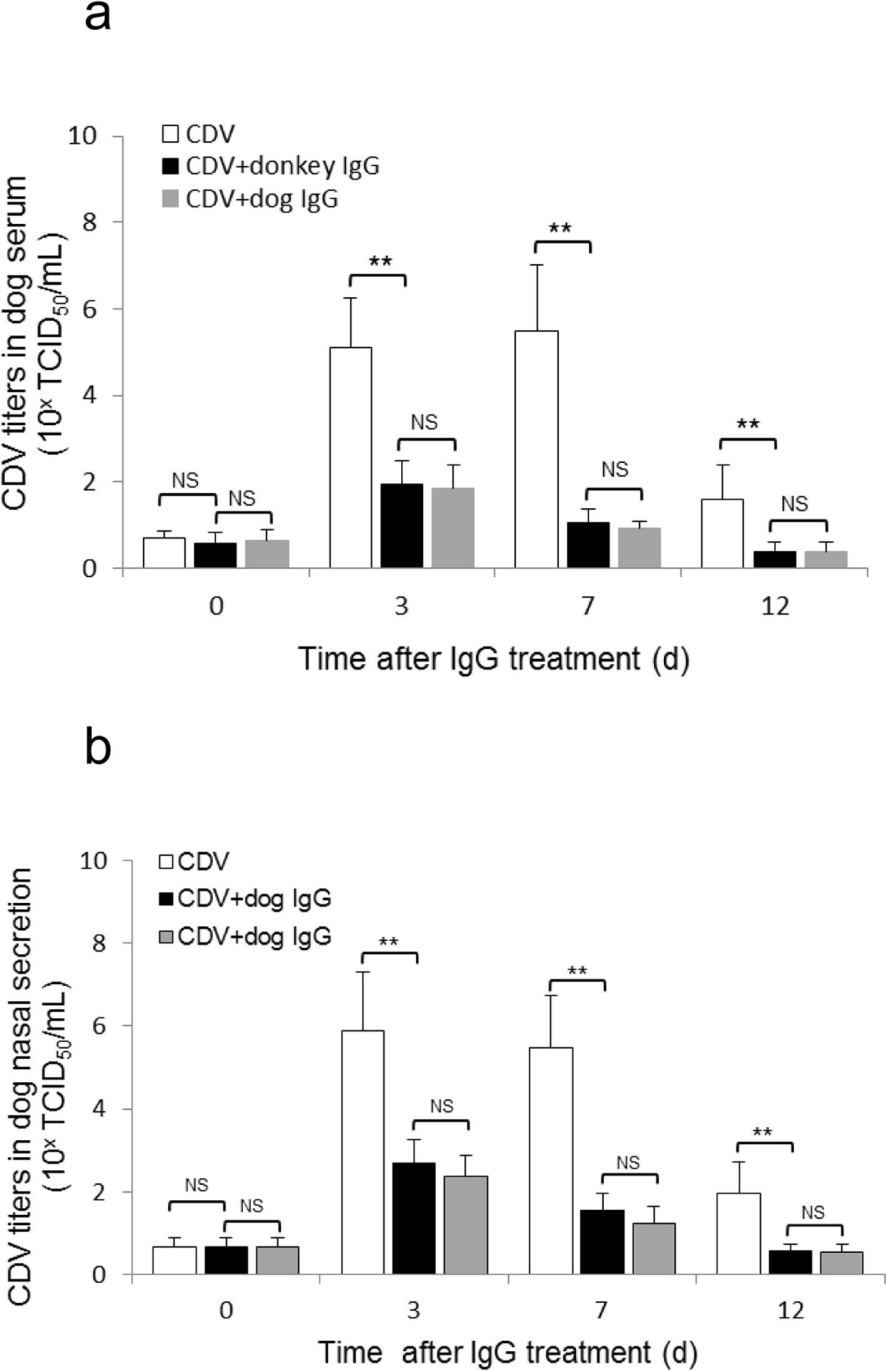
Effects of donkey IgG and dog IgG on CDV replication in CDV-infected dogs. (a) CDV titters in the serum of CDV-infected dogs treated with different IgG at different time (at 0, 3, 7 and 12 d after IgG treatment). (b) CDV titters in the nasal secretion of CDV-infected dogs treated with different IgG at different time (at 0, 3, 7 and 12 d after IgG treatment). The data are presented as mean ± SD. ^*^ *p* < 0.05; ^**^ *p* < 0.01. NS: no significance

To investigate the inhibitory effects of donkey IgG and dog IgG on CDV replication in CDV-infected dogs, the CDV titers in serum and nasal secretions of the dogs in the different time points (at 3, 6, 10, 15 d after infection, i.e. at 0, 3, 7, 12 d after IgG treatment) were analyzed (Fig. 4). We found that before the treatment with IgG, (at 3 d after CDV infection), the titers of CDV in the serum and nasal secretion of three experimental groups were similar (Fig. 5a and b). Moreover, there was no significant difference (*P* > 0.05) although their virus titers were relatively low (Fig. 5a and b). At 3 d after IgG treatment (i.e. at 6 d after CDV infection), CDV titers in the serum and nasal secretion of the two IgG-treated groups were significantly lower than that of IgG-untreated group (CDV group), accounting only 35–40% of the CDV group. However, there was no significant difference between donkey IgG and dog IgG treated groups (Fig. 5a and b). At 7 d after IgG treatment, the CDV titer in the serum and nasal secretion of two IgG-treated groups sharply declined by about 75% relative to the CDV group (Fig. 5a and b). At 12 d after IgG treatment, the CDV titer in both IgG-treated groups continued to decrease when compared with that of the CDV group (Fig. 5a and b). These results indicate that donkey IgG can significantly inhibit CDV replication in CDV-infected dogs, which was considered to be the molecular basis for the therapeutic effect of donkey IgG. It can be seen from the results that the therapeutic effect of donkey IgG is similar to dog IgG, therefore, it was further confirmed that donkey IgG can potentially replace dog IgG in clinical applications.

### No adverse effects exhibited in the dogs after administration of donkey IgG

Before performing the therapeutic effects of donkey IgG on CD, the adverse reactions were analyzed by intramuscular injection of donkey IgG into healthy puppies. It has been observed that the donkey IgG-treated dogs exhibited only mind irritation for 1–2 d after injection, with mild lethargy and slight loss of appetite, without skin eruption, dyspnoea or acute hypersensitivity reactions. The body temperature of the dogs slightly increased by a range of 0.4 to 1.0 °C within 3 h of IgG administration (data not shown). These results indicate that donkey IgG is relatively safe in the treatment of canine distemper.

## Discussion

CD is one of the most common yet fatal infectious diseases in pet clinic [5]. Due to the special status of dogs as pets, people’s attitude towards treating dog diseases is different from other livestock and poultry. Because pet dog is the partner of human beings, the cost of treatment has not been thoroughly taken into consideration when treating dog’s disease, therefore, the treatment of these dogs is highly valued. On the contrary, for other livestock and poultry, if the cost of treatment exceeds its own value, the treatment will be abandoned. Therefore, the treatment of CDV, canine parvovirus (CPV) and other canine infectious diseases occupies the important positions in the clinical practice of pets [32]. For a long time, many attempts have been made to treat CD using antiviral drugs such as ribavirin which is extensively used to treat human viral diseases [33, 34]. However, no obvious therapeutic effect was reported in treatment of CD and CPV [35, 36]. Long term veterinary clinical practice has confirmed that passive immunotherapy with antiserum or antibody is an effective method for the treatment of animal viral infectious diseases [37]. Antibody-mediated passive immunotherapy is a type of immunotherapy in which the therapeutic antibody generated from homologous and heterologous animals are given to the diseased animals to help them fight infectious diseases [38]. So far, the passive immunotherapy using antiserum or antibody has been used for over a century for the treatment of human various diseases [39, 40] since antiserum or antibody can not only block the virus from infecting host cells, but also promote complement-mediated viral neutralization as well as mediating the uptake of pathogen-infected cells by phagocytes [35]. The antibody-mediated passive immunotherapy for CD began in the 1930s [41]. The clinical practice showed that antiserum or antibody could significantly reduce clinical symptoms and prolong the survival period of the diseased dogs [12, 42]. For a long time, the antiserum or antibody used in clinical treatment of canine distemper and canine parvovirus mainly came from dogs [13]. However, in recent years, with the increasing number of dogs and clinical cases, the antiserum or antibody from dogs could not meet the growing market demand, therefore, other large animals, such as horses [18] and pigs [12], have been explored to prepare antiserum or antibodies for future application in the treatment of dog infectious diseases.

In order to expand the source of therapeutic antibodies, in this study we have tried preparing antiserum and antibodies against canine distemper from donkeys, and the therapeutic experiment for the experimental canine distemper was performed using the prepared antibodies. The results showed that the high titer (2^13^) of anti-CDV antiserum and neutralizing antibody (2^6^–2^8^) were obtained by immunizing donkeys with inactivated CD vaccine. The purity of prepared donkey IgG is about 98%. Passive immunotherapy with donkey IgG showed that the mortality of the dogs in donkey-derived IgG treated group is 25% (3/12), significantly lower than that in IgG-untreated dogs (75%, 9/12), indicating that donkey-derived IgG possesses the potent activity to increase the survival rates of CDV-infected dogs. Similarly, the donkey-derived IgG can significantly inhibit CDV replication in CDV-infected dogs (*P* < 0.01). Our results also showed that there is no significant difference between donkey-derived IgG and dog-derived IgG for treatment of CDV-infected dogs in mortality and CDV replication. This result suggests that donkey-derived IgG could potentially substitute dog-derived IgG for CD treatment in pet clinic.

There are some advantages in using donkey to prepare heterologous antibodies to treat canine distemper or other infectious diseases. First of all, donkeys come from a wide range of sources, especially in the vast rural areas of northern China and in Africa, where the number of donkeys is significantly higher than that of horses. In addition, the disease resistance of the donkey is obviously higher than that of the horses, therefore, donkeys seldom suffer from equine infectious diseases, such as equine infectious anemia. In addition, the cost of preparing animal IgG from donkey is relatively low. Therefore, donkey is a potential animal resource for producing heterologous antibodies for passive immunotherapy. However, it should be noted that humoral immune response abilities of different donkeys to CDV-inactivated vaccine were quite different. In the process of immunization, the antibody titers of donkeys with high response ability increased significantly 14 days after the first immunization with CDV-inactivated vaccine, and kept in the end. Therefore, it is necessary to select donkeys with strong humoral immune response ability to prepare antiserum or antibody in the future.

From the passive immunotherapy for the canine distemper, we have learned that the time of administration is the key factor for the therapeutic effects with anti-CDV IgG. We have compared the therapeutic effects at the different time points of the IgG delivery and found that the earlier the time of administration, the better the therapeutic effects (data not shown). If the administration time is delayed until neurological symptoms appear, the therapeutic effect is poor and the sick dog is difficult to recover. This reflects that anti-CDV IgG may have the preventive effect on CD.

In passive immunotherapy with heterologous animal antibodies, it is necessary to consider the immune rejection of the recipient to the heterologous protein. Although there are no strong toxic and side effects in the treatment of CD with donkey-derived IgG, there are also some adverse reactions. These reactions may be related to the IgG purity and the immune rejection of the recipient to the heterologous IgG. In order to solve these problems, the preparation conditions should be optimized to improve the quality of IgG. Furthermore, the IgG should be modified. Currently, the commonly used effective method is to treat IgG with papain, cut off the Fc fragment of IgG to prepare the Fab fragment of IgG, so as to reduce the antigenicity of IgG. To increase therapeutic efficacy of the donkey-derived anti-CDV IgG, the IgG can be chemically modified crosslinking certain cytokines (or chemokines) to stimulate or recruit immune cells to kill infected CDV directly.

## Conclusion

In summary, in this study, by immunizing healthy donkeys with inactivated CDV vaccine and purifying donkey IgG, we have successfully developed the donkey-derived IgG with high-titer neutralizing activity against CDV. The purified IgG, like dog-derived IgG, can inhibit CDV replication in vitro and in vivo, and reduce the mortality of dogs experimentally infected with virulent CDV, suggesting that the donkey-derived anti-CDV IgG could potentially substitute the dog-derived IgG in the pet clinic for CD treatment.

## Methods

### Animals, cells, viruses and dog anti-CDV antibody

Five healthy 4-year-old male donkeys (Dezhou Donkey), weighing 110–130 kg, negative for equine infectious anemia and malleomycosis disease, were provided by a local donkey farm in Hebei Province of China. Thirty-six healthy 3-month-old dogs (Shih-tzu Dog), weighing 1.5–2 kg, negative for CDV, CPV and infectious bronchitis virus (CIBV), were purchased from a local dog farm in Hebei Province of China. All animal experiments were ethically acceptable and approved by the Animal Ethics Committee of Hebei Agricultural University (No. 2017–0012). The CDV (CDV-WH, a virulent strain) was provided by Dr. Weiquan Liu, School of Biology, China Agricultural University. Vero cell line (derived from African green monkey kidney cells), used for amplifying CDV and testing the therapeutic effects of donkey antiserum or IgG against CDV in vitro, were purchased from American Type Culture Collection (ATCC). Dog anti-CDV serum and IgG were prepared in our laboratory.

### Inactivated CDV vaccine preparation

The Vero cells were infected by CDV-WH, the cells were harvested when the cytopathic effects (CPE) of the cells was 85%, after 3 cycles of freeze-thaw treatment, the CDV crude solution was clarified by centrifugation (12,000 rpm, 10 min). The CDV solution was titered by 50% tissue culture infectious dose (TCID_50_) and then mixed with formalin solution (40% formaldehyde) at the ratio of 1000: 1 and incubated at 4 °C on shaker for 24 h to inactivate CDV. Finally, the inactivated CDV solution was mixed with aluminum hydroxide adjuvant at the ratio of 10: 1 to prepare inactivated CDV vaccine. The CDV vaccine was stored at 4 °C. Before use, the vaccine was mixed well by vibration.

### Animal immunization

**Fig. 6:**
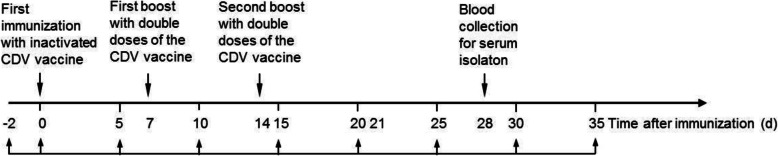
Timeline of immunization of donkeys with CDV vaccine

Five healthy donkeys were raised in the isolated environment and tested for equine infectious anemia with agar diffusion method and malleomycosis with mallein test [43, 44]. After being identified to be negative, the donkeys were immunized by multi-point intramuscular injection of 3 mL of the inactivated CDV vaccine (1.0 × 10^6^ TCID_50_), and then boosted with 6 mL of the inactivated CDV vaccine twice at one-week intervals. Blood samples were collected from the jugular vein before every immunization and the sera were separated from the blood for antibody titeration. Two weeks after the secondary boost, blood was collected (about 1500 mL for a donkey), and serum was separated for the purification of anti-CDV IgG. The timeline of immunization of the donkey is presented in Fig. 6. After the experiment, all the experimental donkeys were kept in isolation for 4 months and then released.

### Antibody titer measurement

Titers of CDV-specific antibodies in the sera of the immunized donkeys were measured by an indirect ELISA using CDV solution as the antigen. Briefly, The CDV solution was diluted 4 times with coating buffer (0.05 mol/L carbonate buffer, pH 9.6). The 96-well plates (Corning Costar, USA) were coated with 200 μL the virus diluent at 4 °C overnight. After blocking with 100 μL of 3% gelatin in PBS containing 0.05% Tween-20 (PBST) at 37 °C for 2 h, 100 μL of two-fold serially diluted donkey serum samples (1, 32, 1, 64 ~ 1, 32,768) were added to the wells, and incubated at 37 °C for 1 h (a negative serum control and a blank control without serum were also set). After washing with PBST, wells were incubated with 100 μL horseradish peroxidase (HRP) -conjugated rabbit anti-donkey IgG (Santa cruz, USA; 1: 20,000) at 37 °C for 1 h. After washing with PBST, 100 μL freshly-prepared 3,3′,3,5′ -tetramethylbenzidine (TMB) solution was added to each well and incubated in the dark at 37 °C for 20–30 min for color development. When the color of the reaction in the wells tends to be stable, 50 μL of 2 mol/L H_2_SO_4_ were then added to each well to stop the reaction, and *OD*_450_ values were read using a microplate reader (Bio-Rad, USA).

### Neutralizing antibody titer determination

One hundred μL of Dulbecco’s Modified Eagle Medium (DMEM) medium containing 5% fetal bovine serum (FBS) was added into each well of 96-well plate, and 100 μL of heat-inactivated (56 °C) donkey serum was added into the first row of wells, and then two-fold diluted on the plates with 3 repeats. Twenty μL of 10^–8.5^ TCID_50_ CDV solution was added into each well and incubated at 37°Cfor 1 h, and then 1 × 10^4^ Vero cells were added into each well and cultured for 2 d. The wells were scored for the presence of CPE. The virus neutralizing titer was determined to be the reciprocal of the highest dilution in which there was no visible CPE.

### IgG isolation and purification

Anti-CDV IgG in donkey serum was isolated by salting out and purified by DEAE-cellulose ion exchange chromatography. Briefly, the donkey serum was diluted with 2 volume of 0.9% NaCl, and then half volume of saturated ammonium sulfate solution was slowly added. After centrifugation (5000×g, 20 min) at 4 °C, the precipitate (globulin) was dissolved with 0.9% NaCl, then one-third volume of saturated ammonium sulfate solution was added and incubated at 4 °C for 30 min. After centrifugation (5000×g, 20 min) at 4 °C, the second precipitate (IgG) was dissolved with 0.9% NaCl. The ammonium sulfate in the sample was removed by Sephadex-G25 gel filtration. The resulting crude IgG was purified by ethylenediamine tetraacetic acid (DEAE)-cellulose ion exchange chromatography. IgG purity was identified with acetate membrane electrophoresis.

### CDV infection in dogs

Thirty-six healthy dogs were randomly divided into 3 groups, the CDV group (*n* = 12), the CDV + dog IgG group (*n =* 12) and the CDV + donkey IgG group (*n =* 12). The dogs in all groups were kept in a conditioned environment (with temperature of 20–25 °C and humidity of 45–55%) for 3 days. The dogs were allowed free access to food and water at all time. The dogs in all groups were injected intramuscularly with hydrocortisone (15 mg/mLethanol), and then infected with 3 mL of CDV virulent strain (1 × 10^6^ TCID_50_) by oral administration (2 mL) and nasal drip (1 mL). The clinical symptoms were checked day by day after the infection. The suspected puppies with CD were identified by clinical symptoms and detecting CDV in the serum with colloidal gold assay using Test Kit for Antigen to Canine Distemper Virus (Ybscience, Shanghai, China). At the end of the experiment, uncured dogs were euthanized by intravenous injection of 5% pentobarbital (1.5 mL /kg body weight) and 10% KCl (0.3 mL /kg body weight).

### Therapeutic effect analysis

When the CDV-infected dogs in the three groups showed the fever and positive CDV test in blood (about 3–4 d after infection), the dogs in the CDV + dog-IgG and CDV + donkey-IgG groups were treated with donkey-derived and dog-derived anti-CDV IgG, respectively, by intramuscular injection (30 mg/kg body weight) three times, 48 h apart. While the dogs in the CDV group were treated with the same dosage of healthy donkey negative serum. After injection of the IgG, the clinical symptoms of the dogs were observed every day, the body temperature was measured, blood was collected at the different time (0, 5, 10, 15 d) after CDV infection for CDV titer measurement. The CDV titers in blood and nasal secretions of the dogs were measured. The survival rates of CDV-infected dogs were analyzed by Graphpad Prism software. The timeline of CDV infection and IgG treatment is presented in Fig. 4.

### Virus titration

The titer of CDV in the blood and nasal secretion of CDV-infected dogs were measured with TCID_50_ method in Vero cells. The dog blood was collected and the serum was isolated and diluted with PBS. The dog nasal secretion was collected and dissolved with PBS and collected supernatant after centrifugation. The collected blood and processed nasal samples were 10-fold diluted on a 96-well plate, each dilution was repeated for 10 times. One hundred μL of different diluted samples were added to each well of 96-well plate with 95% confluent Vero cells. The cells on the plates were cultured at 37 °C for 72 h, the cell morphology was observed under the microscope, the number of wells which showed CPE was recorded, and TCID_50_ was calculated according to Reed-Muench formula.

### Statistics

The significance of differences between experimental groups was evaluated by one-way analysis of variance (ANOVA) with Dunnett’s post-comparison test for multiple groups and Student’s t-test was used for a single comparison of the two groups, respectively. The survival rates were calculated using the Kaplan Meier method and Log-Rank method was use for significance test of survival.

## Data Availability

The datasets used and/or analysed during the current study are available from the corresponding author on reasonable request.

## Abbreviations

CD: Canine distemper
CDV: Canine distemper virus
ELISA: Enzyme-linked immunosorbent assay
TCID_50_: Tissue culture infectious dose
IgG: Immunoglobulin G
CPV: Canine parvovirus
CIBV: Infectious bronchitis virus
ATCC: American Type Culture Collection
CPE: Cytopathic effects
HRP: horseradish peroxidase
TMB: 3,3′,3,5′-tetramethylbenzidine
DMEM: Dulbecco’s Modified Eagle Medium
FBS: Fetal bovine serum
DEAE: Ethylenediamine tetraacetic acid
